# Molecular detection of *Dirofilaria* spp. and host blood-meal identification in the *Simulium turgaicum* complex (Diptera: Simuliidae) in the Aras River Basin, northwestern Iran

**DOI:** 10.1186/s13071-020-04432-4

**Published:** 2020-11-04

**Authors:** Fariba Khanzadeh, Samad Khaghaninia, Naseh Maleki-Ravasan, Mona Koosha, Mohammad Ali Oshaghi

**Affiliations:** 1 Department of Plant Protection, Faculty of Agriculture, University of Tabriz, Tehran, Iran; 2 Department of Parasitology, Pasteur Institute of Iran, Tehran, Iran; 3 Research Center for Emerging and Reemerging Infectious Diseases, Pasteur Institute of Iran, Tehran, Iran; 4 Department of Medical Entomology and Vector Control, School of Public Health, Tehran University of Medical Sciences (TUMS), Tehran, Iran

**Keywords:** *Simulium turgaicum* complex, Vector incrimination, *Dirofilaria immitis*, *D. repens*, Blood meal

## Abstract

**Background:**

Blackflies (Diptera: Simuliidae) are known as effective vectors of human and animal pathogens, worldwide. We have already indicated that some individuals in the *Simulium turgaicum* complex are annoying pests of humans and livestock in the Aras River Basin, Iran. However, there is no evidence of host preference and their possible vectorial role in the region. This study was conducted to capture the *S. turgaicum* (*s.l*.), to identify their host blood-meals, and to examine their potential involvement in the circulation of zoonotic microfilariae in the study areas.

**Methods:**

Adult blackflies of the *S. turgaicum* complex were bimonthly trapped with insect net in four ecotopes (humans/animals outdoors, irrigation canals, lands along the river, as well as rice and alfalfa farms) of ten villages (Gholibaiglou, Gungormaz, Hamrahlou, Hasanlou, Khetay, Khomarlou, Larijan, Mohammad Salehlou, Parvizkhanlou and Qarloujeh) of the Aras River Basin. A highly sensitive and specific nested PCR assay was used for detection of filarial nematodes in *S. turgaicum* (*s.l*.), using nuclear *18S* rDNA-ITS1 markers. The sources of blood meals of engorged specimens were determined using multiplex and conventional *cytb* PCR assays.

**Results:**

A total of 2754 females of *S. turgaicum* (*s.l*.) were collected. The DNA of filarial parasites was detected in 6 (0.62%) of 960 randomly examined individuals. Sequence analysis of 420 base pairs of *18S* rDNA-ITS1 genes identified *Dirofilaria* spp. including 5 *D. immitis* and 1 *D. repens*. Importantly, all filarial positive specimens have been captured from humans and animals outdoors. *Cytb*-PCR assays showed that in all ecotypes studied, members of the *S. turgaicum* complex had preferably fed on humans, dogs, bovids, and birds, respectively.

**Conclusions:**

To the best of our knowledge, this is the first report of *D. immitis*/*D. repens* detection in blackflies. Results showed that *S. turgaicum* (*s.l*.) was the most abundant (97%) and anthropophilic (45%) blackfly in all studied ecotypes/villages and that DNA of *Dirofilaria* spp. was detected in the flies taken from six villages. Dirofilariasis is a common zoonosis between humans and carnivores, with mosquitoes (Culicidae) as the principal vectors. Further investigations are needed to demonstrate that blackflies are actual vectors of *Dirofilaria* in the studied region.
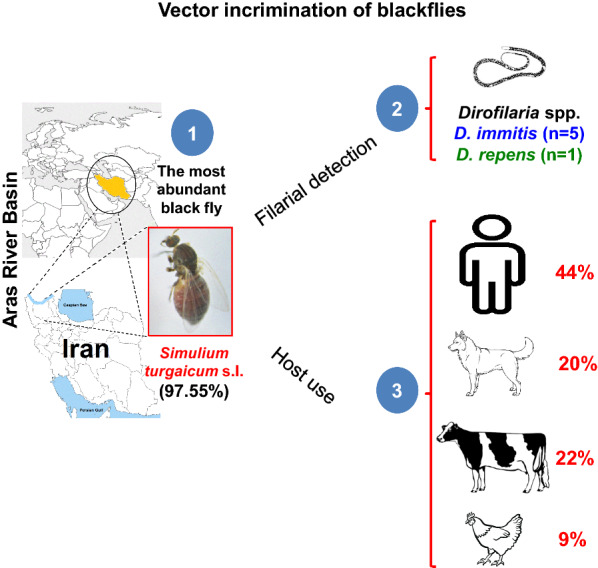

## Background

Blackflies, buffalo gnats or turkey gnats (Diptera: Simuliidae) are known to be effective vectors among arthropod groups since they are vigorous in circulation of 28 vertebrate pathogens and parasites, besides the causal agent of human onchocerciasis is vectored by 25 *Simulium* spp. [1, 2]. Potentially they are responsible for transmitting pathogenic agents ranging from protozoan parasites (*n* = 17) to filarial nematodes (*n* = 15) and several arboviruses among numerous hosts [2]. In this spectrum, only two human diseases, onchocerciasis and mansonellosis, are caused by simuliid-borne filarial nematodes [1].

Onchocerciasis is caused by the parasitic worm *Onchocerca volvulus* and may lead to itching, disfiguring dermatitis, and eye lesions, including permanent blindness in humans. Although most infected people reside in Africa south of the Sahara, the disease is widespread to some areas in Latin America and Yemen, as well. Estimates in 2017 exposed that there were 21 million *O. volvulus* infections worldwide; 14.6 million of the infected people with skin disease and 1.15 million with vision loss [3].

The pathology of mansonellosis, caused by *Mansonella ozzardi*, appears to be mild [4]. The disease is restricted to the New World tropics where the disease is vectored *via* at least five simuliid species and biting midges of the genus *Culicoides* [5].

In addition to being proficient vectors of important disease agents, blackflies are annoying pests of humans and animals due to their swarming and long-term blood-sucking behaviors resulting in significant economic losses in sectors of society from agriculture, animal husbandry, and forestry to tourism, and entertainment [6–10]. These flies are among the few arthropods that have the potential to kill animals by their extreme blood-feeding and serious toxemia (simuliotoxicosis) originated from the saliva [11, 12].

According to the last inventory of blackflies, a total of 2348 species including 2331 living and 17 fossil have been recorded as valid species worldwide [13]. The Palaearctic Region covers the most described blackflies species, with 33%, followed by the Oriental (17%), and Neotropical (16%) Regions [13]. The Palaearctic *Simulium* subgenus *Wilhelmia* comprises 31 living species throughout Europe, Asia and Africa that are acknowledged as noteworthy and dominant pests of humans and livestock [8, 9, 14–17].

*Simulium turgaicum* is a species complex in the *S. equinum* species-group in the subgenus *Wilhelmia*. We recently showed with detailed morphological and molecular studies that *S. turgaicum* comprises three well-supported lineages in Iran [9]. The divergence within *S. turgaicum* has previously been shown by other researchers, as well [16–18].

Simuliids are reported to be the most insidious insects in the Aras River Basin, so that farmers and ranchers have had to wear beekeeping hats or alter their working hours from day to night. In this region, blackflies enter farmers’ eyes, ears, mouth, and nose throughout the day. However, in the late hours of the day and in crowded places, blackflies fly recklessly against all parts of humans and animals (our personal observations). The *S. turgaicum* complex dominates the blackfly community in the Aras River Basin, accounting for 97% of the specimens in our previous study [9].

There is little knowledge of the potential vectorial role of blackflies in Iran where no documentation exists on their role in the circulation of anthroponotic or zoonotic pathogens in the country. Although to date *Simulium* spp. have been described as vectors of *Dirofilaria ursi*, a parasite of American and Asian bears [19], to the best of our knowledge, no studies have been carried out regarding blackflies as vectors of mosaic zoonoses of *D. immitis*/*D. repens*. This study aimed to (i) determine the abundance of *S. turgaicum* complex in the Aras River basin of Iran, (ii) identify their host blood meals through PCR assays, and (iii) examine the potential involvement of the *S. turgaicum* complex in the circulation of zoonotic microfilariae in the region.

## Methods

### Study area, blackfly collection and identification

The study was conducted in the south of the Aras River, in Khoda-Afarin County, East Azarbaijan Province, in northwestern Iran. Due to the climatic conditions, water resources are abundant in this region so that the main occupations of the population are farming and animal husbandry. Adult blackflies were bimonthly trapped with a conventional insect net in four ecotopes (humans/animals outdoors, irrigation canals, lands along the Aras River, and rice and alfalfa fields) of 10 villages (Gholibaiglou, Gungormaz, Hamrahlou, Hasanlou, Khetay, Khomarlou, Larijan, Mohammad Salehlou, Parvizkhanlou and Qarloujeh) from May to October 2016–2018. Specimens were stored in 70% ethanol at 4 °C until investigation. Morphological identifications of the specimens were achieved using the key by Crosskey [20]. The specimens with empty and blood-fed abdomens were applied for filarial and mammalian hosts’ genome analysis, respectively.

### DNA extraction, filarial species detection and sequencing

A subset (31–71%) of the specimens with empty abdomen (unfed or fully gravid) from each site was selected for filarial genome analysis. The selected specimens included representatives of all the blackflies caught per time and per net in the analysis. Adult blackflies were separately rinsed twice with 70% ethanol at 10,000 × *rpm* for 2 min. The whole bodies of dehydrated flies were pulverized using narrow metal pestles in 1.5 ml microtubes. The total genomic DNA was extracted using the Collins et al. [21] method. A highly sensitive and specific nested PCR assay developed by Tang et al. [22] was applied for detection of potential filarial nematodes in blackflies using nuclear *18S* rDNA-ITS1 markers. These primers could amplify conserved regions of filarial *18S*, *5.8S* rDNA, and ITS1 from *O. volvulus*, *Wuchereria bancrofti*, *Loa loa*, *Mansonella ozzardi*, *M. perstans*, *M. streptocerca*, *Brugia malayi*, *B. timori*, and *Dirofilaria* spp.

Originally, primers of UNI-1R: 5ʹ-CGC AGC TAG CTG CGT TCT TCA TCG-3ʹ and FIL-1F: 5ʹ-GTG CTG TAA CCA TTA CCG AAA GG-3ʹ were applied to amplify a 712–771 bp of a partial sequence of the target loci. The PCR product of the first step was used as a template for the second step. In this step, the primers of FIL-2F: 5'-GGT GAA CCT GCG GAA GGA TC-3' and FIL-2R: 5ʹ-TGC TTA TTA AGT CTA CTT AA-3ʹ were used to amplify a 286–420 bp fragment. The PCR was performed in a total volume of 25 μl containing 5 μl (~0.5 μg) of genomic DNA for the first step of nested PCR, and 2.5 μl of PCR product for the second step, 12.5 μl of *Taq* DNA polymerase 2× master mix red (Ampliqon, Odense, Denmark), and 1 μl of each primer (10 mM), which was supplemented by double distilled water, in an automated Thermocycler (Analytik Jena FlexCycler; Midland, Ontario, Canada).

The PCR conditions were set as an initial denaturation at 94 °C for 7 min, followed by (nest-one) 40 cycles of denaturation at 94 °C for 20 s, annealing at 60 °C for 20 s, and extension at 72 °C for 30 s, or (nest-two) 35 cycles of denaturation at 94 °C for 20 s, annealing at 50 °C for 20 s, and extension at 72 °C for 20 s with a final extension at 72 °C for 7 min. PCR products were visualized with 1% agarose gel electrophoresis, followed by GreenViewer staining and imaging, using a UV transilluminator. Fruitful amplicons were sequenced in both directions *via* Genetic Codon Company (Tehran, Iran), using the same primers as for amplification.

The respective sequences of 6 specimens are deposited in GenBank. Twenty-two reference sequences belonging to various nematodes were retrieved from GenBank and used for multiple sequence alignment and relationship inference by MEGA X [23].

### Blood-meal analysis of blackflies

Two multiplex and conventional *cytb*-PCR methods were employed to investigate host use by the blackflies, according to the protocol modified from Lahiff et al. [24], Parodi et al. [25], Ngo & Kramer [26], and Chang et al. [27]. Based on our preliminary study and knowledge of the animal species in the study area, 4 sets of primers (avian, bovine, dog and human) were used to determine the source of blood meals. Genomic DNA from blood-fed female blackflies was extracted using the Collins et al. [21] method. DNA from dog, bovine, hen, and human blood was set as positive controls, and DNA of male blackflies, as well as water, was used as negative controls. Reagents in the multiplex PCR assay were the same as those for the filarial detection; however, the thermal conditions for initial denaturation were at 94 °C for 10 min, continued by 35 cycles of denaturation at 95 °C for 30 s, annealing at 53 °C for 40 s, and extension at 72 °C for 30 s, followed by a final extension at 72 °C for 8 min. The specific PCR for each avian, bovine, dog, and human host corresponded with annealing temperatures of 55 °C, 58 °C, 56 °C and 64 °C, respectively. PCR products from the two types of assays were analyzed by 2% agarose gel electrophoresis stained by GreenViewer dye (Parstous, Mashhad, Iran) and photographed on a UV transilluminator.

## Results

### Entomological and parasitological findings

**Fig. 1:**
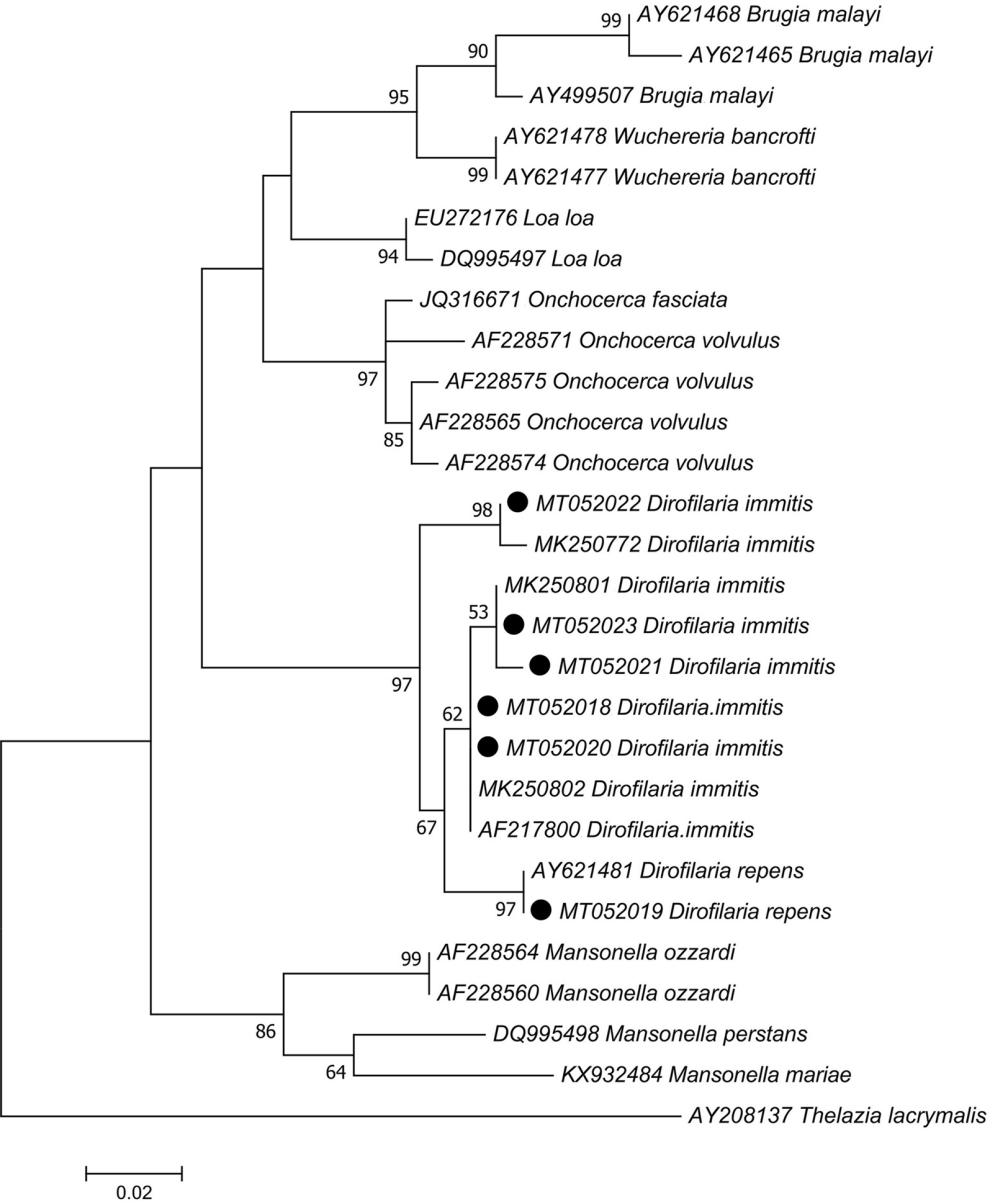
Maximum likelihood tree showing the relationships between the *18S* rDNA-ITS1 sequences obtained in this study (solid circles) and other filarial nematodes retrieved from GenBank. *Thelazia lacrymalis* was designated as outgroup. The numbers at the branch points are bootstrap values based on 1000 replicates. Bootstrap values less than 50% are hidden. The scale-bar measures evolutionary distance in substitutions per nucleotide

Large numbers of blackflies were captured from the study areas, of which 97% including 2754 females and 300 males were morphologically identified as the *Simulium turgaicum* complex. Representative specimens of each ecotope in each village were selected for detection of filarial nematodes in *S. turgaicum* (*s.l*.) The DNA of filarial parasites was detected in 6 (0.62%) of 960 individuals (Table 1). Importantly, all filarial positive specimens have been captured from humans and animals outdoors. Amplicons from the positive specimens were purified and sequenced. Sequence analysis of ~ 420 bp of *18S* rDNA and ITS1 regions identified *Dirofilaria* spp. including 5 *D. immitis* and 1 *D. repens*. Five sequences of *D. immitis* (MT052018, MT052020-23) showed 99.05–100% identity with the isolates reported from Thailand (MK250772, MK250801, MK250802). A sequence of *D. repens* (MT052019) displayed 99.47% similarity with a sequence reported from Thailand (AY621481). Relationships analysis using *18S* rDNA and ITS1 sequences confirmed that the specimen clustered with the *Dirofilaria* spp. lineage, with high bootstrap values (Fig. 1).

**Table 1:** Females of *Simulium turgaicum* complex captured in Aras River Basin, Iran, and tested for *Dirofilaria* spp. infection

Village	Ecotope (*n*)	No. of collected specimens	No. of specimens used for filer detection (%)	No. of infected (%)	*Dirofilaria* species	GenBank ID
Deim-Qarloujeh	Irrigation canals in rural areas (*n* = 513)	513	220 (43)	1 (0.45)	*D. repens*	MT052019
Gholibaiglou	Lands along the Aras River (*n* = 57), rice and alfalfa fields (*n* = 43)	100	40 (40)	–	–	–
Gungormaz	Lands along the Aras River (*n* = 75), humans and animals outdoors (*n* = 105)	180	60 (33)	1 (1.67)	*D. immitis*	MT052020
Hamrahlou	Lands along the Aras River (*n* = 178), humans and animals outdoors (*n* = 142)	320	100 (31)	–	–	–
Hasanlou	Lands along the Aras River (*n* = 18), rice and alfalfa fields (*n* = 7)	25	10 (40)	–	–	–
Khetay	Humans and animals outdoors (*n* = 396)	396	110 (28)	1 (0.91)	*D. immitis*	MT052023
Khomarlou	Lands along the Aras River (*n* = 142)	142	50 (35)	1 (2)	*D. immitis*	MT052018
Larijan	Lands along the Aras River (*n* = 72), humans and animals outdoors (*n* = 33)	105	50 (48)	1 (2)	*D. immitis*	MT052021
Parvizkhanlou	Alfalfa field (*n* = 42)	42	30 (71)	–	–	–
Qarloujeh	Rice and alfalfa fields (*n* = 453), humans and animals outdoors (*n* = 478)	931	290 (31)	1 (0.34)	*D. immitis*	MT052022
Total		2754	960 (35)	6 (0.625)	–	–

*Note*: The data on the males of *S. turgaicum* (*s.l*.) and the remaining 3% collected blackflies are not shown here

*Abbreviation*: n, total number of specimens

### Vertebrate hosts

The sources of the blood meals were determined for 113 blood-fed specimens of the *S. turgaicum* complex trapped from different ecotypes of five villages, i.e. Gholibaiglou, Gungormaz, Hamrahlou, Khetay and Qarloujeh, in the Aras River Basin. Although blood-fed flies were present in all ecotopes studied, most of them were trapped from humans/animals outdoors, an epidemiologically important ecotope. Selected primers for avian, bovine, dog, and human hosts yielded 508, 271, 153, and 228 bp products, respectively, in both multiplex and specific *cytb*-PCR assays. Multiplex PCR results revealed four patterns of single (*n* = 14), double (*n* = 23), triple (*n* = 8) and quadruple (*n* = 1) blood sources among 46 specimens of *S. turgaicum* (*s.l*.) The method revealed blood-meal sources as human (45%), dog (24%), bovine (22%), and bird (9%). Nine out of 46 (19.6%) specimens had at least mixed dog and human blood. Among the 113 blood-fed specimens tested by conventional PCR, 50 (44%) fed on humans, 26 (23%) on bovids, 22 (20%) on dogs, and 15 (13%) on birds (Table 2). Feeding on humans varied among the populations of *S. turgaicum* (*s.l*.), from 30% to 51%.

**Table 2:** Identification of vertebrate hosts of the *Simulium turgaicum* complex based on the host *cytb* gene in ingested blood meals by multiplex and conventional PCR assays in the Aras River Basin, Iran

Village	Ecotope (No. of blood-fed specimens)	No. of blood-fed specimens in each village	Multiplex PCR		Host-specific PCR				
			*n*	Hosts	*n*	Hosts			
						B	H	C	D
Qarloujeh	Humans/animals outdoors (*n* = 23); irrigation canals (*n* = 14); lands along the river (*n* =17); rice and alfalfa fields (*n* = 19)	73	18	DH (*n* = 1); CD (*n* = 2); BD (*n* = 2); CH (*n* = 9); BH (*n* = 2); BCD (*n* = 1); BCDH (*n* = 1)	73	7	37	13	16
Hamrahlou	Humans/animals outdoors (*n* = 2)	2	–	–	2	–	–	2	–
Khetay	Humans/animals outdoors (*n* = 15)	15	15	H (*n* = 8); D (*n* = 1); DH (*n* = 2); CD (*n* = 3); BCD (*n* = 1)	15	4	5	2	4
Gungormaz	Humans/animals outdoors (*n* = 13)	13	11	H (*n* = 4); CD (*n* = 2); BCD (*n* = 1); BDH (*n* = 2); CDH (*n* = 2)	13	4	4	3	2
Gholibaiglou	Humans/animals outdoors (*n* = 10)	10	2	D (*n* = 1); BDH (*n* = 1)	10	–	4	6	–
Total		113	46	46	113	15	50	26	22

*Abbreviations*: B, bird; C, bovine; D, dog; H, human

## Discussion

Blackflies have been the focus of many studies since they are strong blood-feeders and competent vectors of human and animal disease agents. However, to date, they have not been implicated in the infection and possible transmission of *D. immitis* or *D. repens*. We found a high prevalence of *S. turgaicum* (*s.l*.) in the region, with blood meals primarily from humans and dogs, and with DNA of the microfilariae of both species in the specimens with empty abdomens which are unfed or fully gravid. Detection of filarial DNA in blackflies with empty abdomens indicates considerable age or survival which is epidemiologically thought-provoking indicating that the parasite is still present following the blood-digestion process and may evolve to its infective stage. The second point should be confirmed in future studies by confirming the vitality of the nematodes or the developmental stages of the microfilariae in the epidemiologically important parts of the fly’s body.

Dirofilariasis is largely caused by two parasites, *D.* (*Dirofilaria*) *immitis* (Leidy, 1856) and *D.* (*Nochtiella*) *repens* (Railliet & Henry, 1911) (Spirurida: Onchocercidae), in humans and carnivores, with distinct clinical and epidemiological features. *Dirofilaria immitis* is responsible for severe cardiopulmonary dirofilariasis, whereas *D. repens* causes non-pathogenic subcutaneous dirofilariasis in canids and felids. Accordingly, *D. immitis* and *D. repens* are globally regarded as human pulmonary and hypodermic/ophthalmic dirofilariasis, respectively [28, 29]. Herein we detected both nematodes in the study areas; however, their clinical manifestations need to be determined in detail in the future.

Successful development of the *Dirofilaria* species depends on an intermediate mosquito species and a vertebrate as a definitive host [30]. Many species of culicid mosquitoes in the genera *Aedes*, *Anopheles*, *Armigeres*, *Coquillettidia*, *Culex*, *Culiseta*, *Mansonia* and *Psorophora* allow the development of both *D. immitis* and *D. repens* [31–55] which may transmit carrying worms to available vertebrates. *Dirofilaria immitis*, can parasitize many canines and felines in tropical, subtropical, and temperate regions worldwide [56]. However, filarial infections of dog, cat, fox, wolf, coyote, and least weasel by *D. repens* have been documented only in Old World countries [56]. Humans are not appropriate hosts for *Dirofilaria* spp.; however, among mammalian hosts, dogs are the most important reservoir host for human infection of both species of *Dirofilaria* [57].

Human and animal dirofilariasis has been documented in 11 of the 31 provinces of Iran. In two main foci of the country (southern regions of the Aras River: East Azerbaijan and Ardebil Provinces), the highest frequencies of dirofilariasis caused by *D. immitis* were 36.8% in dogs, 57.1% in jackals, 50% in foxes, 50% in wolves, and 0.8% in cats, and by *D. repens*, 60.8% in dogs and 10% in jackals [48, 58]. Fourteen cases of human subcutaneous, ocular, testicular, and pulmonary dirofilariasis have been reported in the country [58]. The infective third-stage larvae of *D. immitis* and *Setaria labiatopapillosa* (Alessandrini) (Spirurida: Onchocercidae) were, respectively, reported in *Cx. theileri* and *An. maculipennis* [48].

Even if the presence of DNA of these parasites in the blackflies is suggestive, further studies are needed to demonstrate that blackflies are true vectors of *D. immitis*/*D. repens*. *Simulium turgaicum* (*s.l*.) was the most widespread blackfly in all ecotypes studied of the ten villages of Khoda-Afarin County. The *Dirofilaria* spp. infection rate in *S. turgaicum* (*s.l*.) (0.625%) was significantly lower compared to those observed in culicid mosquitoes (0.85–10%) [29]. These flies frequently feed on humans and dogs and potentially could pick up *Dirofilaria* spp. The possibility of blackflies as vectors of the *Dirofilaria* in this study is reinforced by studies showing that *D. ursi* Yamaguti, 1941 is transmitted by blackflies to black bears in North America and Japan [59, 60]. Likewise, in support of the vectorial activities of simuliids in Iran, a nodular eye lesion of human onchocerciasis caused by *O. lupi* and the involvement of blackflies in its transmission has been reported from center of the country [61].

Complex interactions between intrinsic and extrinsic factors influence vector competence. Intrinsic factors, such as genetics and physiology, as well as behavioral traits, may govern an insect’s ability to acquire, support development, and transmit pathogenic agents. Extrinsic factors include the populations of the host reservoir and their activity patterns, climatic conditions, genetic variability in infectivity of pathogens, and even gut microorganisms [62–67]. Although blackflies are recognized as one of the most important medical and veterinary groups of arthropods due to their vector competence, there is a little information on their biology and ecology in Iran. This study is the first step for issuing public health policies and blackfly control campaigns in Iran, as well as those interested in studying the factors affecting vector eligibility.

Development of filarial nematodes to the third larval stage in the intermediate insect vector is essential for successful transmission to the definitive host. Microfilaria such as *D. ursi* develops to the third larval stage in the Malpighian tubules of the corresponding vector [68]. However, vector incrimination of blackflies relies on the presence of L3 worms in the head capsules of the female insects [68]. Future studies should be conducted to locate microfilariae in the Malpighian tubules and head capsules of the females of *S. turgaicum* (*s.l*.)

The literature shows that *D. immitis* and *D. repens* can infect many species of mammals varying in both quantity and quality [29]. Dissimilarity in susceptibility to parasites is common in multi‐host, multi‐parasite collections, and can have profound significance for ecology and evolution in these systems [69, 70]. This may lead to heterogeneous and asymmetric transmission among and between host species [71–74]. The results of the present study show that insects other than Culicidae mosquitoes may be involved in establishing a cycle of dirofilariasis in the region. Since both *D. immitis* and *D. repens* can infect many species of mammals and can be transmitted by various vector species of mosquitoes and blackflies, they exhibit poor host specificity in terms of definitive and intermediate hosts [75]. These findings challenge our ability to recognize, forecast, and handle dirofilariasis dynamics in the region.

The results of this study revealed that *S. turgaicum* (*s.l*.) has diverse host preferences and feeds on humans, dogs, bovids and birds. A majority (69%) of the specimens fed on humans and/or dogs. This may be related to host availability in the region and could have important epidemiological consequences. Dogs are the main reservoir of *Dirofilaria* spp., and if a blackfly prefers to feed on humans and dogs, then the probability of becoming infected and then transmitting the parasite to a human will be increased. However, to incriminate *S. turgaicum* (*s.l*.) as an actual vector of *D. immitis* and *D. repens*, more information on the biological and ecological factors that influence the transmission of these parasites in the studied region should be provided. These are included, but not limited to, parous rates, daily and seasonal fluctuations of biting activities (especially of parous females), preference for human body parts, longevity, flight range, and gonotrophic cycle [76].

## Conclusions

Gathering site-specific information for vectors is a vital stage in implementation of vector control measures. Our study provides preliminary information on dirofilariasis in an active focus in northwestern Iran. We identified the dominant blackfly taxon in the study area and addressed issues of anthropophily and presence of *Dirofilaira* spp. in specimens with an empty abdomen. We are currently surveying nematode distributions in epidemiologically important parts of the bodies of blackflies. These findings could be used in epidemiological studies and strategic planning for the control of dirofilariasis in the region.

## Data Availability

The datasets utilized in drawing the conclusions of this article are included in the article.

## Abbreviations

*18S* rRNA: *18S* Ribosomal RNA
*cytb*: Cytochrome b
ITS1: Internal transcribed spacer 1
*s.l*.: *sensu lato*
